# Experimental and Theoretical Studies of the Electronic Band Structure of Bulk and Atomically Thin Mo_1–*x*_W*_x_*Se_2_ Alloys

**DOI:** 10.1021/acsomega.1c02788

**Published:** 2021-07-15

**Authors:** Jan Kopaczek, Tomasz Woźniak, Magdalena Tamulewicz-Szwajkowska, Szymon J. Zelewski, Jarosław Serafińczuk, Paweł Scharoch, Robert Kudrawiec

**Affiliations:** † Department of Semiconductor Materials Engineering, Faculty of Fundamental Problems of Technology, Wroclaw University of Science and Technology, Wybrzeże Wyspiańskiego 27, 50-370 Wrocław, Poland; ‡ Department of Nanometrology, Wroclaw University of Science and Technology, Janiszewskiego 11/17, 50-372 Wroclaw, Poland

## Abstract

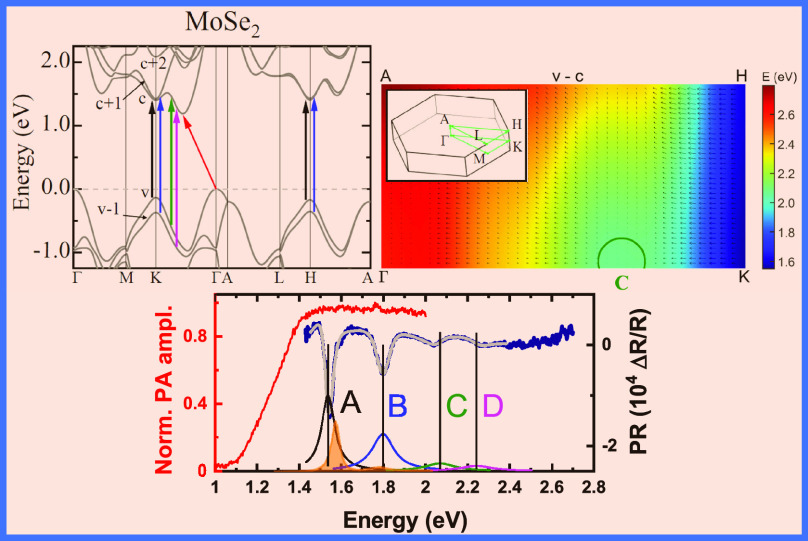

We present studies focused on the evolution of the electronic band structure of the Mo_1–*x*_W*_x_*Se_2_ alloy with the tungsten content, which was conducted by combining experimental and theoretical methods. Employed spectroscopic techniques, namely, photoreflectance, photoacoustic spectroscopy, and photoluminescence, allowed observing indirect and direct transitions at high and beyond high-symmetry points of the Brillouin zone (BZ). Two excitons (A and B) associated with the K point of the BZ were observed together with other optical transitions (C and D) related to band nesting. Moreover, we have also identified the indirect transition for the studied crystals. Obtained energies for all transitions were tracked with a tungsten content and compared with results of calculations performed within density functional theory. Furthermore, based on the mentioned comparison, optical transitions were assigned to specific regions of the BZ. Finally, we have obtained bowing parameters for experimentally observed features, for, i.e., thin-film samples: *b*(A) = 0.13 ± 0.03 eV, *b*(B) = 0.14 ± 0.03 eV, *b*(C) = 0.044 ± 0.008 eV, and *b*(D) = 0.010 ± 0.003 eV.

## Introduction

In the last decade, the interest in studying bulk and especially atomically thin transition metal dichalcogenides (TMDs) has substantially increased. This growth of interest eventuates from the feasibility of thinning down the bulk van der Waals (vdW) crystals to single-atomic layers, the concept initially proposed by Geim et al. for obtaining graphene.^1,2^ The mentioned reduction of the original crystal thickness leads to significant changes in the electrical and optical properties of TMDs. One of the most substantial alterations is the change of the fundamental band gap from an indirect to a direct one,^3−6^ allowing atomically thin crystals to be used in nanoelectronics and optoelectronics.^7−10^ To extend the application possibilities of TMD materials, alloying them can be exploited for tuning their band gap in a wide spectral range, the approach commonly used for III–V semiconductors.^11−15^ To date, studies of binary crystals greatly outnumber articles about TMD alloys, among which only a few are devoted to optical research of the Mo_1–*x*_W*_x_*Se_2_ alloy.^16−18^ In those works, compositional dependencies of energy only for A and B excitons are studied. For the A exciton in the atomically thin crystal, the bowing parameters *b* equal to 0.14^17^ and 0.151 eV^18^ were obtained, whereas for A and B excitons, single-crystal values of 0.16 and 0.12 eV^16^ were determined. The said bowing parameter indicates the non-linearity of transition energies versus the composition of the crystal, which for highly mismatched alloys^19,20^ such as, e.g., GaNAs or GaAsBi, are as high as 7.5 eV (for the N content above 8%)^21^ and 1.74 eV,^22^ respectively. The value of parameter *b* of a given alloy AB_1–*x*_C*_x_* could also be compared to the difference between the energy gap of constituent semiconductors, i.e., Δ*E* = *E*_g_(AC) – *E*_g_(AB), since when *b* > Δ*E*, the energy gap of AB_1–*x*_C*_x_*, the alloy for some content *x*, can be smaller than the *E*_g_(AB). Such a case was shown experimentally for Mo_1–*x*_W*_x_*Se_2_ in refs (17) and (18), despite the slight difference between molybdenum and tungsten in terms of electronegativity and atomic size. The observed behavior was explained based on the partial charge density calculations.^17^ In the case of the optical transition energetically above A and B excitons, the transitions at high-symmetry points of the Brillouin zone (BZ) and band nesting were studied only for binary TMD compounds.^23−29^ Those studies were performed for bulk and atomically thin crystals, primarily using optical modulation spectroscopy and spectroscopic ellipsometry.

The modulation techniques, due to their derivative-like character, allow studying, with great precision, optical transitions that correspond to specific *k*-points of the BZ.^24,30^ Such experimental methods, when combined with theoretical calculations of the electronic band structure, mainly based on density functional theory (DFT), are the perfect approach for unambiguous assignment of the optical transition to a given *k*-point of the BZ of novel semiconductor materials. This approach was applied for group IV,^31,32^ III–V,^14,33^ and II–VI^34,35^ semiconductor alloys and, at present, for, i.e., binary TMD compounds^26,27,29^ as well as alloys.^36,17,37^ It was shown that, beyond A and B excitons at the K point of the BZ, studied mainly by photoluminescence (PL), other spectral features are visible.^29^ These features, i.e., optical transitions, measured also by modulation techniques such as photoreflectance (PR) or piezoreflectance (PzR), were assigned to the band nesting region and higher energetically lying bands at high-symmetry points of the BZ.^24,38^

Here, we report on experimental and theoretical studies of the electronic band structure of ∼100 μm-thick and atomically thin Mo_1–*x*_W*_x_*Se_2_ alloys. The experimental studies of optical transitions were performed at room temperature by PR and PL techniques used for single-crystal and monolayer samples, respectively, whereas to obtain electronic band structures of studied alloys, calculations within DFT were conducted. Based on the two above-mentioned approaches, the evolution of the band structure with the tungsten content was tracked and compared. These dependencies, namely, the energy of transitions versus tungsten concentration, were fitted with linear and quadratic relationships to determine the nonzero bowing parameter. In the case of a single crystal, the said comparison was performed for all experimentally observed transitions, including the indirect one obtained by photoacoustic spectroscopy (PA). In contrast, the A exciton for the monolayer sample was studied solely experimentally.

## Sample Preparation

The studied 2H-vdW crystals (99.9999% confirmed purity), with different compositions synthesized by the flux zone method, were obtained from a 2D Semiconductors company. The homogeneity of atom concentration and lack of phase separation were confirmed by the company.^39^ The thickness of the macroscopically studied Mo_1–*x*_W*_x_*Se_2_ thin films was prepared to be about 100 μm. Furthermore, the atomically thin samples were obtained by the conventional mechanical exfoliation technique.^40,41^ The studied monolayer samples are shown in Figures S1–S6.

## Experimental Methods

To obtain the tungsten content in the Mo_1–*x*_W*_x_*Se_2_ alloy, we have made the X-ray diffraction (XRD) measurements using a Phillips MRD X-ray diffractometer system with the parallel beam optics and Cu_Kα1_ radiation of λ = 1.540597 Å. The Θ–2Θ scans (with a 0.1 mm slit in the diffracted beam optic) were employed to collect XRD spectra. The details of XRD studies are presented in the section “XRD analysis” in the Supporting Information.

The PR spectra, i.e., the energy dependencies of the relative changes in the reflection coefficient, were obtained at room temperature in a so-called “bright configuration” experimental setup.^42^ In the PR technique, the mechanically modulated pump beam, i.e., laser line (CW 405 nm), was directed onto a sample, causing the variation of a built-in electric field at the surface and, as a result, evoking changes in the reflection coefficient. These changes were afterward probed by the light beam emitted from a halogen lamp. The probe beam, reflected from the sample, was directed into the monochromator and finally detected; here, we used a 0.55 m focal-length monochromator and a Si p–i–n photodiode. Phase-sensitive detection was utilized using a lock-in amplifier.

The PL spectra for monolayer samples were obtained with the use of a thermoelectrically cooled Si spectrometer. The excitation beam, i.e., 405 nm laser line, was focused (a spot size of ∼30 μm) onto the monolayer area by a long working distance microscope objective (20× magnification).

For determining the indirect fundamental band gap of the thin-film samples, measurements of the absorption coefficient were performed using PA spectroscopy. The studied crystals were periodically illuminated by a monochromatic light, which leads to the generation of heat due to the non-radiative processes. Since the thermal diffusivity is nonzero, heat was transferred into the gas surrounding the sample, causing pressure oscillations detected by an acoustic transducer (electret microphone). Finally, the AC signal was obtained with phase-sensitive detection.

## Computational Methods

DFT calculations have been performed using the Vienna ab initio simulation package (VASP)^43^ with the projector-augmented wave (PAW) method,^44^ including the spin–orbit interaction and D3 correction for vdW interactions.^45^ The exchange-correlation energy was obtained with the Perdew–Burke–Ernzerhof (PBE) parametrization of the generalized gradient approximation (GGA) functional.^46^ For the BZ integrations, a Gaussian smearing of 0.05 eV was used. A plane-wave basis cutoff of 550 eV and a 12 × 12 × 6 Γ-centered Monkhorst–Pack grid of *k*-points were chosen for pure crystal cases to ensure convergence of the optimized lattice constants up to 0.001 Å. Electronic band structures were calculated with the use of the mBJ-TB09 meta-GGA potential.^47^

For the Mo_1–*x*_W*_x_*Se_2_ alloy, special quasirandom structures (SQSs) were employed to model the substitutional random solid solution.^48^ SQS determination was carried out using ATAT software.^49^ From the total energy convergence tests, 3 × 3 × 1 supercells and a pair cluster radius of 6.4 Å were chosen to model *x* = 0.25, 0.5, and 0.75 crystal compositions. The supercell band structures were unfolded onto the primitive BZ with the use of the BandUP code.^50^

For all structures, the atomic positions and lattice vectors were optimized so that the interatomic forces and stress tensor components are lower than 0.001 eV/Å and 0.1 kbar, respectively.

## Results and Discussion

**Figure 1:**
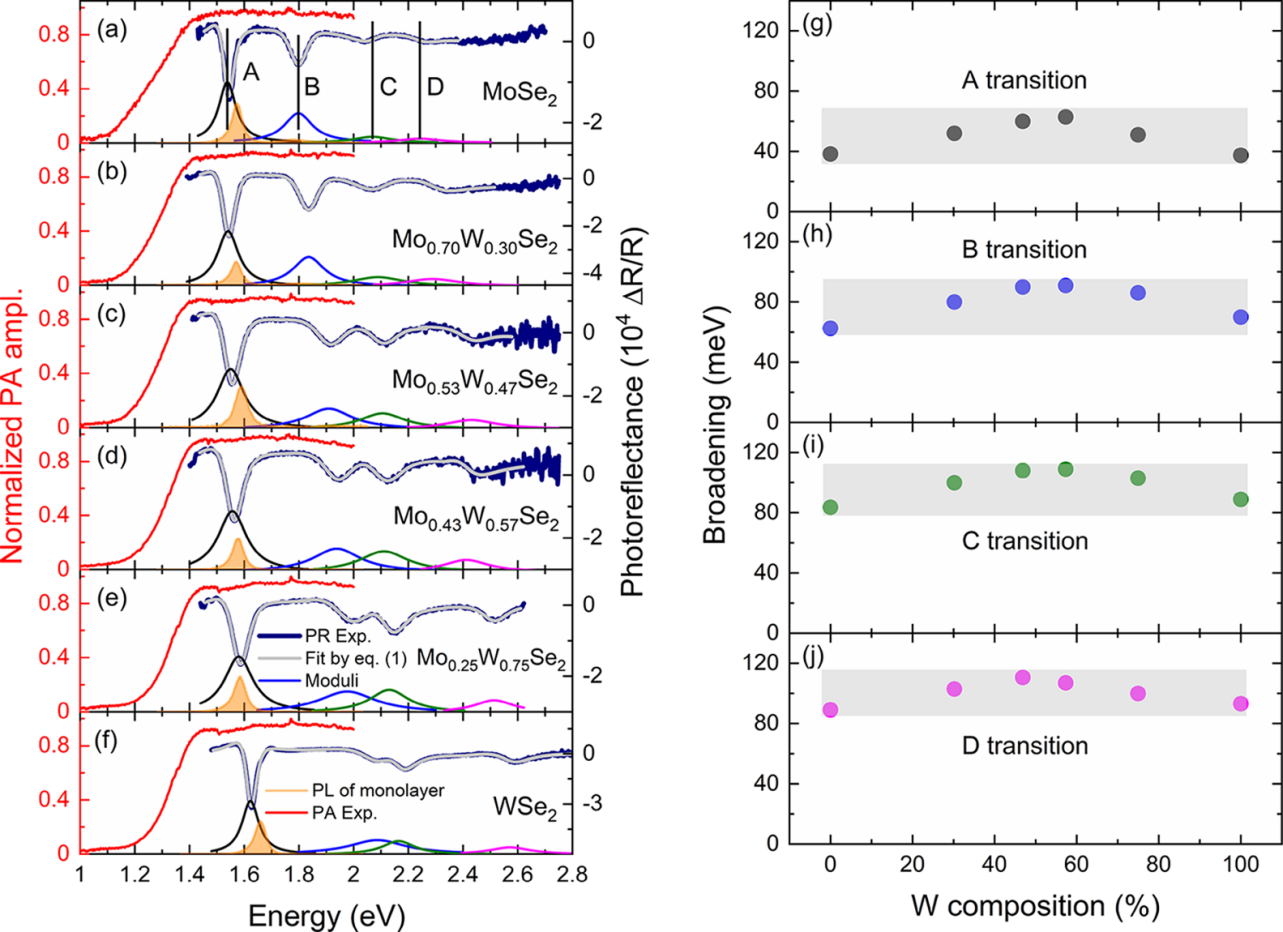
(a–f) PR (navy line) and PA (red line) spectra obtained at room temperature for Mo_1–*x*_W*_x_*Se_2_ bulk crystals. The results of the fitting procedure by eq 1 are marked by a gray line, whereas the color lines plotted at the bottom part of every panel represent the moduli of the given transition. Furthermore, we have shown the normalized PL spectra (orange line) obtained for the monolayer sample. In panels (g–j), the dependencies of broadening parameters versus the tungsten content are plotted.

To track the evolution of the electronic band structure of the Mo_1–*x*_W*_x_*Se_2_ alloy with the tungsten content, we have performed optical measurements at room temperature. The employed experimental techniques, i.e., PR, PA, and PL spectroscopy, allow us to study the optical transitions at specific *k*-points of the BZ. The obtained PR and PA spectra (navy and red lines, respectively) for thin-film samples and PL spectra (orange line) for monolayers are shown in Figure 1a. It can be seen that all measured optical transitions shift to a higher energy with the increase in the tungsten content. The indirect transition marked as “I”, related to the valence band maximum (VBM) at the Γ point and the conduction band minimum (CBM) located halfway between K and Γ points (as identified within DFT calculations), was determined by a so-called knee method from the absorption edge.^51^

The possibility of observing the direct transitions in PR spectra is, among others, due to the presence of van Hove singularities in the function of the joint density of states (JDOS).^52,53^ Furthermore, the condition to be fulfilled to observe the transition at a given energy is ∇_k_(*E*_c_ – *E*_v_) ≈ 0, and such *k*-points at a specific location in the BZ are called the critical points (CPs). When ∇_k_*E*_c_ = ∇_k_*E*_v_ = 0, the CPs are usually located at high-symmetry points of the BZ, while for the band nesting, when ∇_k_*E*_c_ = ∇_k_*E*_v_ ≠ 0, the position of the CP is away from the high-symmetry points.^54^ Two out of all observed direct transitions, namely, A and B, occur at the K point of the BZ, where the second one involves a lower valence band that arises from the spin–orbit interaction.^55,56^ High-energy transitions C and D are associated with the band nestings at the path between K and Γ high-symmetry points.

To obtain the energy and broadening of each optical transition, we have employed a fitting procedure using the Aspnes relation (eq 1), allowing us to retrace features in the relative changes of the reflection coefficient.^57^
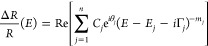
1where *C* and θ are the amplitude and the phase of the signal, whereas *E* and Γ correspond to the energy and the broadening of the transition, respectively. Moreover, the parameter *m* depends on the type of optical transition; in the case of the excitonic one, it is equal to 2. For a better illustration and comparison of transitions, we have plotted for all of them the moduli (colored solid lines in Figure 1) given in eq 2 with the parameters taken from the fitting procedure by the Aspnes relation.
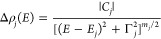
2

The areas under these curves, i.e., moduli, can be treated qualitatively as proportional to the oscillator strength of a given transition. It can be seen in Figure 1a that, at ∼50 meV above the A transition, there is another weak feature A* (change of the slope for the high energy part of the resonance), which can correspond to the H high-symmetry point of the BZ, the excited state of A, or the interlayer excitonic transition.^58−60^ In this work, we do not study the A* transition due to its significantly lower amplitude when compared to the A transition. Additionally, considering the broadening of both transitions, the determination of energy for the A* exciton becomes impossible with reasonable accuracy. Furthermore, the broadening of all studied transitions increases for the ternary compound owing to the compositional fluctuation, as presented in Figure 1b.

**Figure 2:**
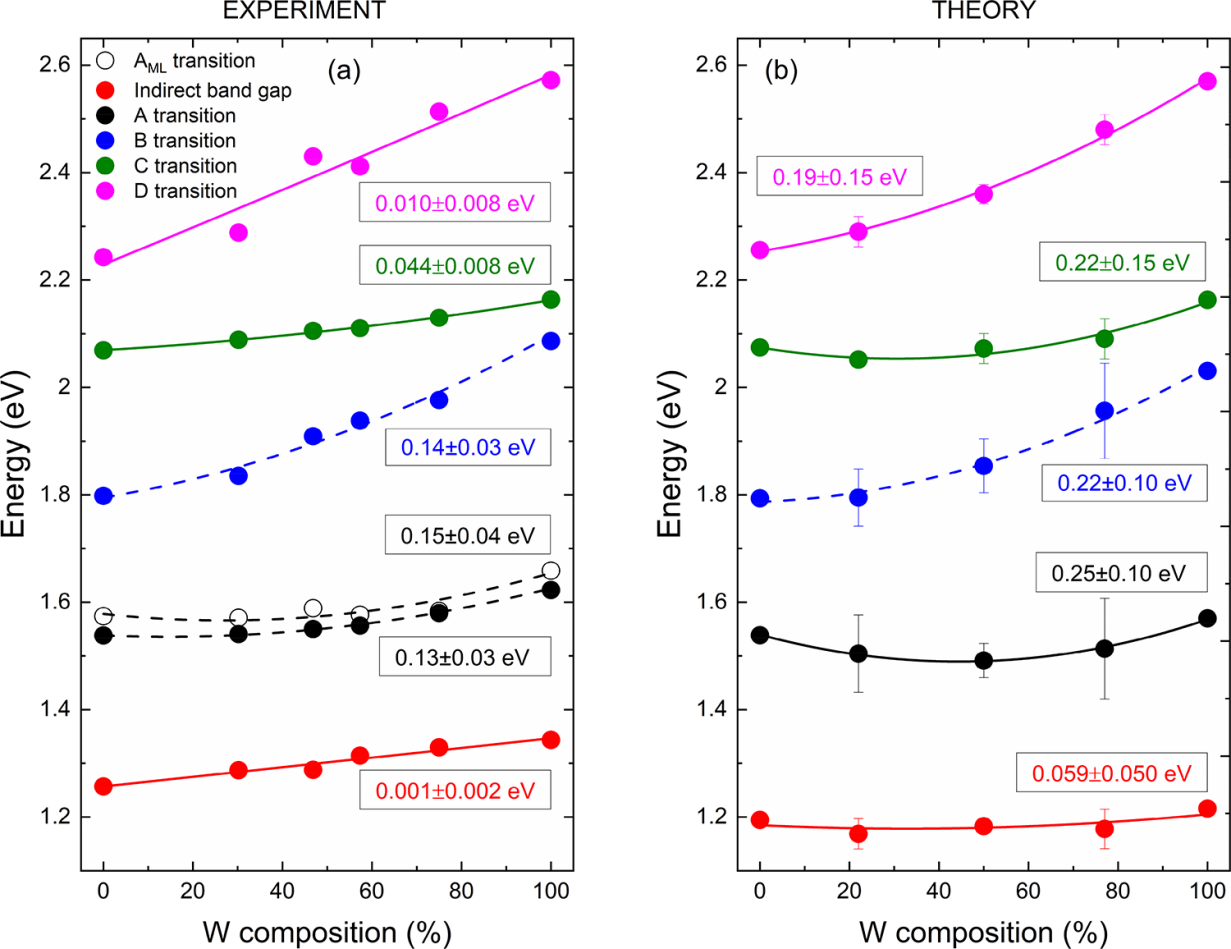
Dependencies of optical transition energies determined in function of the tungsten content for the Mo_1–*x*_W*_x_*Se_2_ alloy determined experimentally (a) and from DFT calculations (b). The parameter, namely, the bowing parameter, describing these relations is shown with its uncertainties.

The energies of the optical transitions extracted from the PR spectra for each sample are plotted as a function of the tungsten content in Figure 2a. It is visible that obtained dependencies for A and B transitions for bulk and the A transition (marked as A_ML_) for monolayer samples are not linear. In contrast, the remaining transition energies, namely, I, C, and D, upshift almost linearly with tungsten concentration.

The strong non-linearity of the A_ML_ transition, despite the similarities between Mo and W atoms in terms of atomic size and electronegativity, was explained by Tongay et al.^17^ on the basis of charge density calculations. These calculations have shown that the states composing CBM, for which the main contribution is from the d*_z_*^2^ orbital of a metal atom, are localized around molybdenum. Moreover, the out-of-plane orientation of the d*_z_*^2^ orbitals leads to their weak coupling between different metals. Since the energy of d*_z_*^2^ is lower for molybdenum atoms, the incorporation of them is responsible for reducing the band gap of the Mo_1–*x*_W*_x_*Se_2_ alloy in a nonlinear manner. A similar explanation of energy bowing of the A transition was presented by Chen et al.^36^ for the Mo_1–*x*_W*_x_*S_2_ alloy. To describe the dependencies of energy with the W content studied in this work, we have fitted them by a linear relation (solid lines) and using eq 3 (dashed lines), which takes into account the non-linearity through the bowing parameter (*b*).
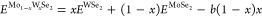
3where *E*^WSe_2_^ and *E*^MoSe_2_^ are the energies of optical transitions for WSe_2_ and MoSe_2_, respectively. The obtained linear coefficients and the bowing parameters are shown in Figure 2 and Table 1. The bowing parameters for A, B, and A_ML_ transitions are in good agreement, including their uncertainties, with previously obtained values: *b*(A) = 0.16 eV and *b*(B) = 0.12 eV^16^ and for the A_ML_ transition equal to 0.14^17^ and 0.151 eV.^18^

**Table 1:** Experimentally and Theoretically Obtained Parameters, i.e., the Bowing Parameter and the Linear Coefficient, Describing Tungsten Content Dependencies of Transition Energies Determined for the Mo_1–*x*_W*_x_*Se_2_ Alloy

	experiment		theory	
transition	*b* [eV]	*a* [meV/%]	*b* [eV]	*a* [meV/%]
A_ML_	0.15 ± 0.04			
I	0.001 ± 0.002	0.90 ± 0.10	0.059 ± 0.050	0.20 ± 0.23
A	0.13 ± 0.03		0.25 ± 0.10	
A_H_			0.21 ± 0.10	
B	0.14 ± 0.03		0.22 ± 0.10	
B_H_			0.19 ± 0.10	
C	0.044 ± 0.008	0.94 ± 0.10	0.22 ± 0.15	0.85 ± 0.35
D	0.010 ± 0.008	3.15 ± 0.33	0.19 ± 0.15	3.21 ± 0.33

It can be seen that the bowing of energy of the remaining transitions is negligible, and with good approximation, these dependencies can be described by linear coefficients. This finding can be explained by the larger, compared to the A transition, contribution of d*_z_*^2^_–*y*_^2^ and d*_xy_* orbitals to the points of the BZ corresponding to C, D, and I transitions.^61^ It is in line with the previously published interpretation in ref (17) since the in-plane orientation of d*_z_*^2^_–*y*_^2^ and d*_xy_* orbitals leads to their stronger coupling for Mo and W atoms and results in a more linear shift of given bands in the BZ.

**Figure 3:**
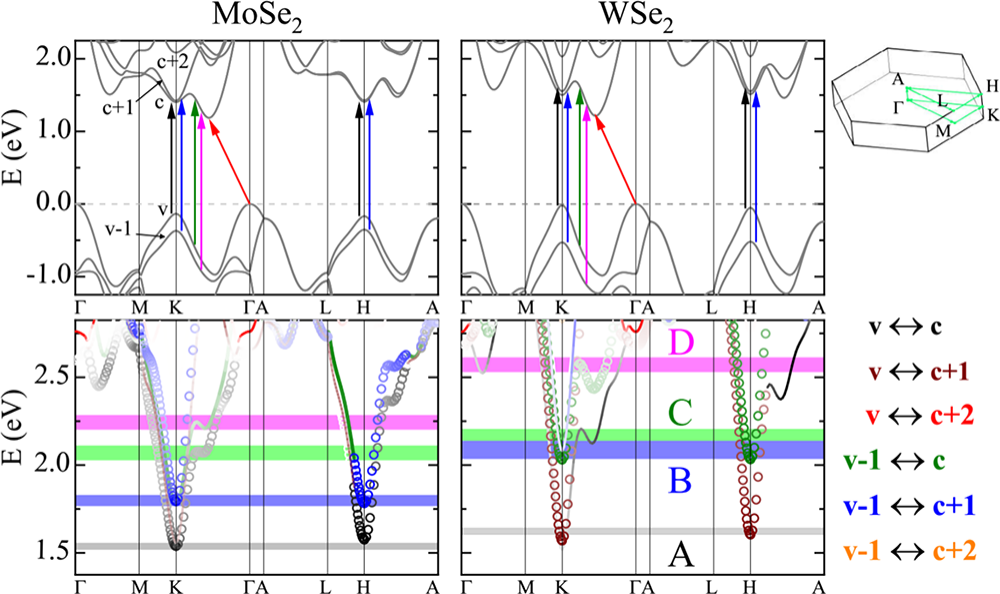
Band structures (top) and direct gaps (bottom) of MoSe_2_ (left) and WSe_2_ (right) with a scissor correction of 140 meV applied to the conduction bands. Experimentally observed transitions are marked with vertical arrows in band structures. On the bottom panels, the energetic separation between bands corresponding to A and B transitions is plotted with circles, while for the remaining transitions, they are plotted with lines. The intensity of the color is proportional to the oscillator strength of the transition. Horizontal bars indicate the experimentally obtained energies of transitions, while their broadenings are represented by the width of the bars.

For the purpose of assigning the optical transitions to specific bands and *k*-points of the BZ, we have compared experimentally and theoretically obtained parameters describing the energy evolution of optical transitions with varying tungsten contents. Moreover, the mentioned PR signal assignment to transitions between bands was performed, taking into account their spin-layer polarization.^30,62,63^ To determine the theoretical parameters, we have calculated the electronic band structure of the Mo_1–*x*_W*_x_*Se_2_ alloy, with different W contents, as described in the Computational Methods section. The used here D3 vdW correction that is relevant for layered crystals, together with the mBJ-TB09 potential, has been shown to work successfully for TMDs.^30,58,64,65^ Furthermore, to reproduce the random distribution of cation (metal) atoms on crystal sites, we used the SQS approach as it was effectively applied to study semiconductor alloys.^15,48,66−68^ The calculated electronic band structures of MoSe_2_ and WSe_2_ are presented on the top panel of Figure 3. We consider here primarily the lowest/highest energetically located conduction/valence bands labeled by *c*/*v* letters. It can be seen in Figure 3 (top panel) that the bands at K and H points of the BZ, i.e., *c*, *c* + 1, *v*, and *v* – 1, are split due to interlayer and spin–orbit interactions.^55^ This splitting together with spin-layer polarization of bands results in two bright optical transitions, labeled as A and B (black and blue arrows). For the molybdenum diselenide crystal, the A and B transitions are from *v* to *c* and from *v* – 1 to *c* + 1 bands, whereas for WSe_2_, the mentioned transitions involve *v* → *c* + 1 and *v* – 1 → *c* bands, respectively, according to the spin-layer polarization of the bands at the K point. Moreover, at a higher energy, we have observed two other direct optical transitions (green and purple arrows). They are located in the out-of-high symmetry points of the BZ, between K and Γ points, where conduction and valence bands are parallel, i.e., band nesting points.

**Figure 4:**
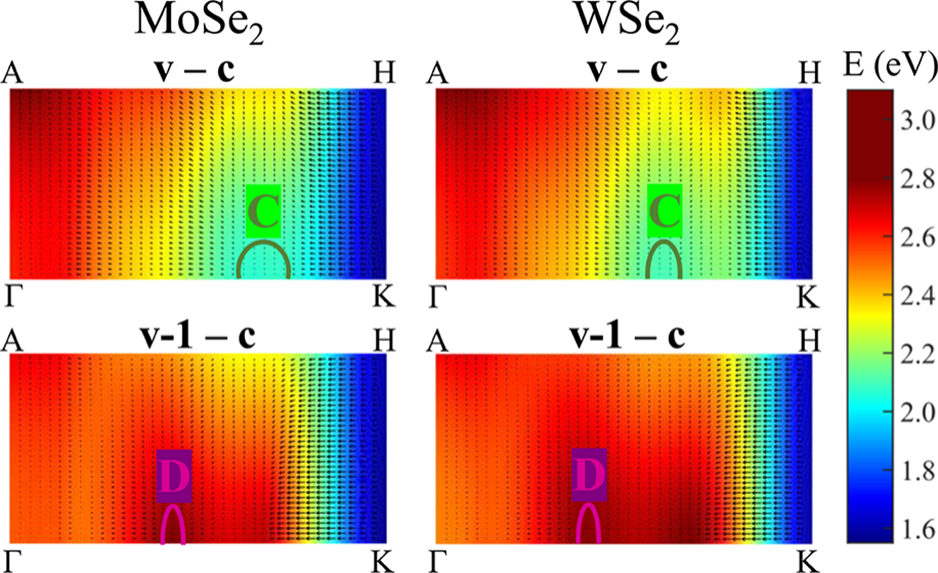
Energy difference between *c* and *v* (−1) bands and its gradient for MoSe_2_ and WSe_2_ on the ΓKHA plane in the BZ. Band nesting regions corresponding to C and D transitions are marked olive and magenta, respectively.

The calculated oscillator strengths of the transitions between different bands are shown in Figure 3 (bottom panel) together with experimental results depicted as horizontal bars, whose widths correspond to the broadening of the optical transitions observed in PR spectra. It can be seen that both K and H high-symmetry points of the BZ may contribute to the features labeled A and B, visible in Figure 1. Nevertheless, it is not possible to distinguish these contributions due to the slight energy difference of transitions at K and H points in comparison to their broadening. Considering the optical transitions at the band nesting points, it is visible that their oscillator strengths are lower (brighter colors in Figure 3, top panel) than for A and B features. Despite the relatively low oscillator strengths of C and D transitions, they are visible in PR spectra due to the contribution from extended *k*-space regions on ΓKHA planes. These areas are marked with olive (C transition) and magenta (D transition) lines in Figure 4 for MoSe_2_ and WSe_2_.

The calculated transition energies in the function of the tungsten content are presented in Figure 2b. All the energies were upshifted by 140 meV for clarity and fitted with the linear or quadratic dependencies, in the same way as in the case of experimental trends in Figure 2a. The uncertainties of energies at intermediate *x* values come from the splitting of electronic bands, which is a characteristic of random crystals modeling by the SQS method^14,48,66^ (see the unfolded band structure of Mo_0.5_W_0.5_Se_2_ in Figure S9).

To confirm the nature of and primarily to assign the features observed in PR spectra to given points in the BZ, we have finally compared the experimentally and theoretically obtained parameters describing dependencies of optical transitions energies on the tungsten content. In Table 1, these parameters are shown with their uncertainties. The mentioned comparison validates the assignment presented in the previous paragraphs as follows: for MoSe_2_, the A exciton involves the transition from the *v* to the *c* band at the K point in the BZ, the B transition is from the *v* – 1 to the *c* + 1 band at the K point, the C transition is from the *v* to the *c* band at the path between K and Γ points, the D transition is from the *v* – 1 to the *c* band at the path between K and Γ points, and the I transition is from the *v* band at the Γ point to the *c* band minimum located at the path between K and Γ points; for WSe_2_, the A transition is from the *v* to the *c* + 1 band at the K point, the B transition is from the *v* – 1 to the *c* band at the K point, and the C, D, and I transitions were assigned in the *k*-space in the same way like for MoSe_2_. Furthermore, a significant finding from the theoretical analysis is a similarity of bowing parameters obtained for A and A_H_ and also for B and B_H_ transitions. This result suggests that the features labeled A/B and observed in PR spectra may be composed of transitions from K and H points in the BZ. It is also worth noticing that the values of the bowing parameter for the A transition obtained for thin-film and monolayer samples are comparable, taking into account their uncertainties. Moreover, it can be concluded that the bowing parameters obtained here for direct transitions are much lower than for III–V highly mismatched alloys as well as for alloys that do not belong to these groups of materials, like GaInAs (0.477 eV) or GaAsSb (1.43 eV).^69^

## Conclusions

In this work, we have studied the evolution of the electronic band structure of the Mo_1–*x*_W*_x_*Se_2_ alloy with the tungsten content by experimental and theoretical methods. To experimentally track the changes at high and out-of-high symmetry points of the BZ, we have employed PR, PL, and PA spectroscopy methods, allowing us to obtain the energy of indirect and direct optical transitions. The obtained bowing parameters for indirect (I) and direct (C and D) at the band nesting point transition are negligible, i.e., hundreds of eV. In contrast, the values of the bowing parameters for the A and B transition are equal to 0.13 and 0.14 eV, respectively. To assign the experimentally observed features, we have calculated the electronic band structure for studied alloys within DFT. Besides two well-known excitons, namely, A and B, we have identified two other band nesting transitions related to out-of-high symmetry points of the BZ located between the K and Γ point. The presented results give new insight into the understanding of the band structure evolution in TMD alloys in a wide spectral range, especially in the band nesting regions.

## Supporting Information Available

Optical images of studied monolayer samples with the images of their emission; the result of the AFM measurement of sample thickness; XRD spectra from planes (002), (004), (006), and (0010) for studied crystals; and the band structure of MoSe_2_ and WSe_2_ and the unfolded band structure of Mo_0.5_W_0.5_Se_2_ (PDF)
