# Supplementary material for: Experimental and Theoretical Studies of the Electronic Band Structure of Bulk and Atomically Thin Mo1–xWxSe2 Alloys
Source: ACS Omega. 2021 Jul 15;6(30):19893–900. doi: 10.1021/acsomega.1c02788 (PMC8340422; doi:10.1021/acsomega.1c02788)
Supplement: Supplementary file 1 — ao1c02788_si_001.pdf [file ao1c02788_si_001.pdf]

Supplementary material for:
Experimental and theoretical studies of the electronic band structure of bulk and atomically thin $Mo_{1-x}W_xSe_2$ alloy

*Jan Kopaczek[1, a)], *Tomasz Woźniak[1], Magdalena Tamulewicz-Szwajkowska[2], Szymon J. Zelewski[1], Jarosław Serafińczuk[2], Paweł Scharoch[1] and Robert Kudrawiec[1]

[1]Department of Semiconductor Materials Engineering, Faculty of Fundamental Problems of Technology, Wroclaw University of Science and Technology,
Wybrzeże Wyspiańskiego 27, 50-370 Wrocław, Poland
[2]Department of Nanometrology, Wroclaw University of Science and Technology, Janiszewskiego 11/17, 50-372, Wroclaw, Poland
* Indicates that these authors contributed equally to this work.
[a)] Corresponding author: jan.kopaczek@pwr.edu.pl

I. Monolayer samples

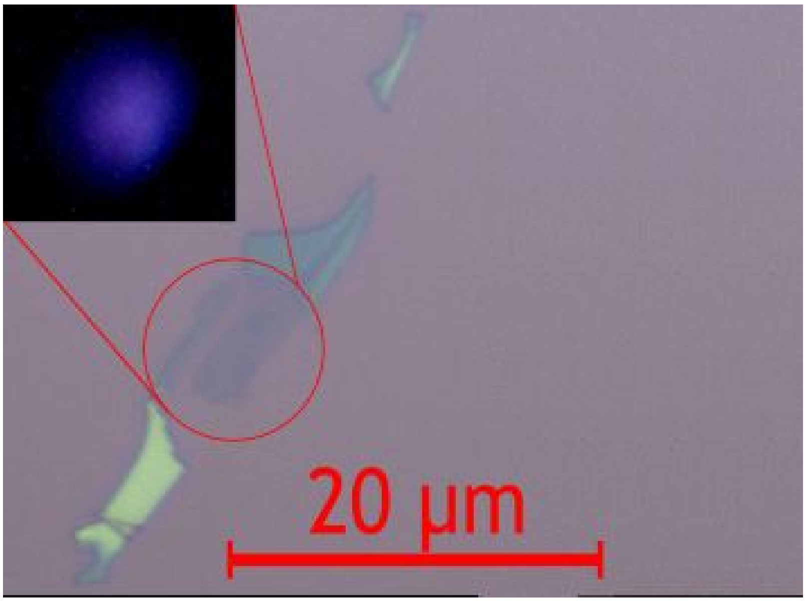

Figure S1. Optical image of the studied $MoSe_2$ monolayer sample together with the picture of emission.

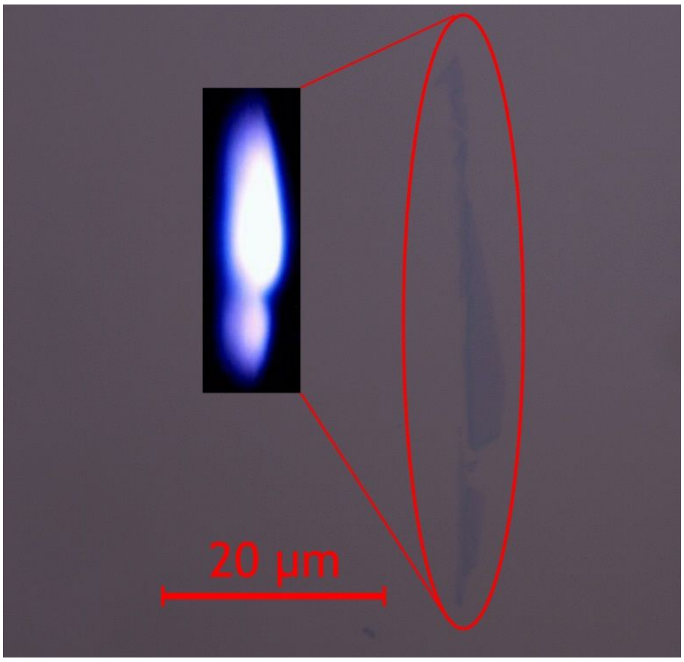

Figure S2. Optical image of the studied $Mo_{0.70}W_{0.30}Se_2$ monolayer sample together with the picture of emission.

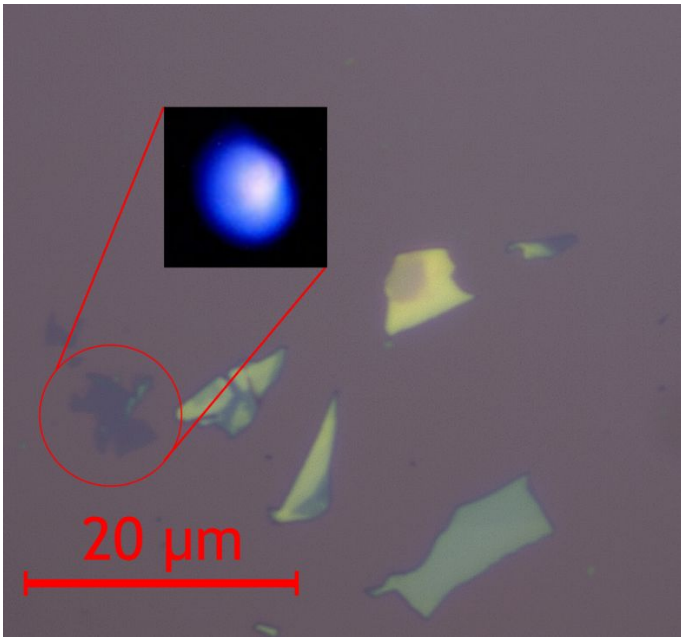

Figure S3. Optical image of the studied $Mo_{0.53}W_{0.47}Se_2$ monolayer sample together with the picture of emission.

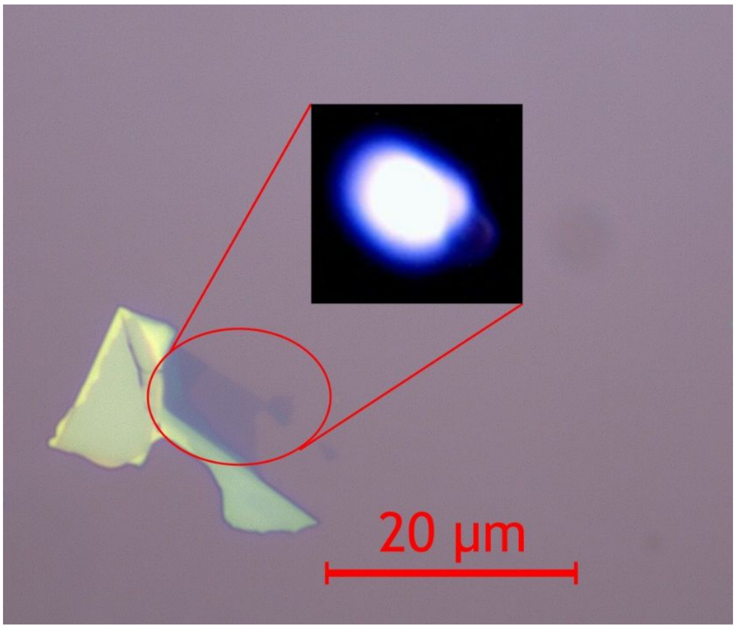

Figure S4. Optical image of the studied $Mo_{0.43}W_{0.57}Se_2$ monolayer sample together with the picture of emission.

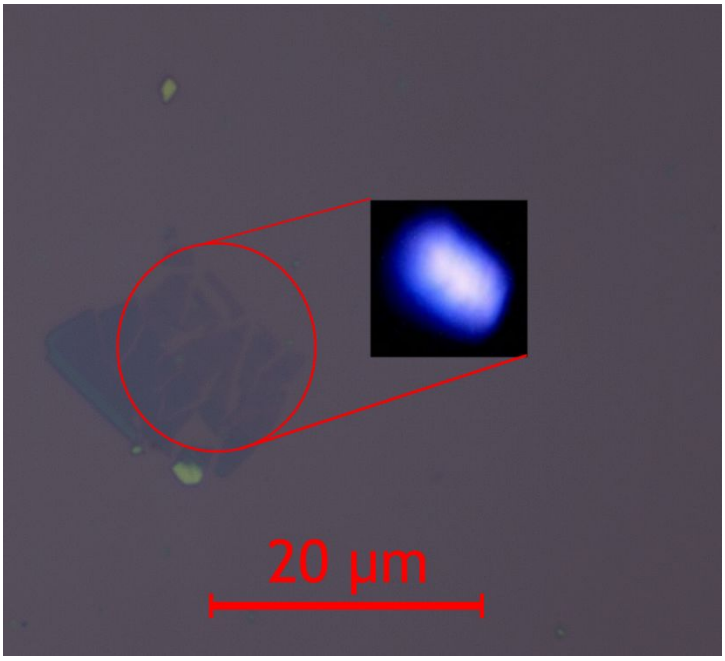

Figure S5. Optical image of the studied $Mo_{0.25}W_{0.75}Se_2$ monolayer sample together with the picture of emission.

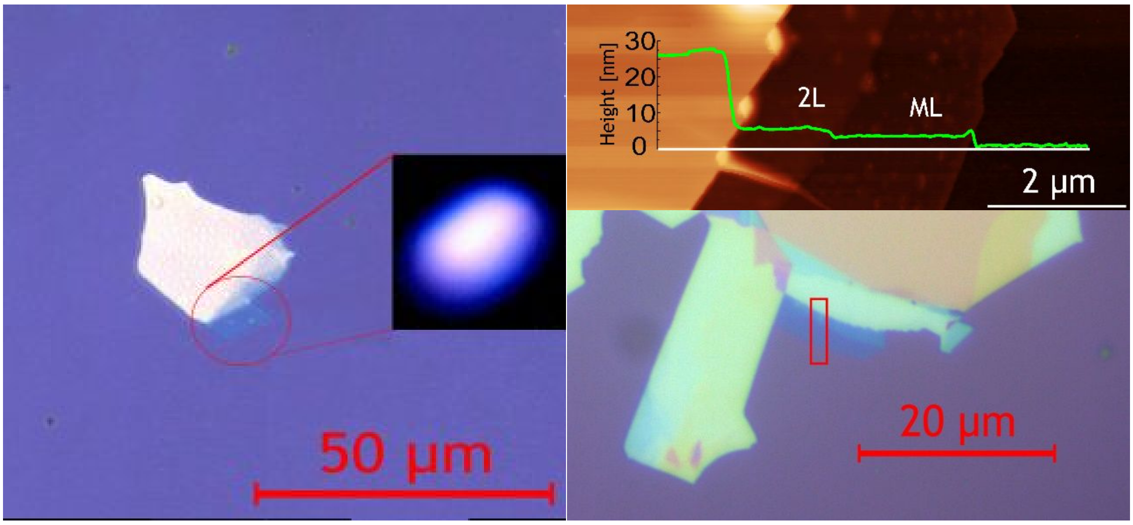

Figure S6. Optical images of the studied WSe_2 monolayer sample together with the picture of emission. Moreover, directly on one of the sample result of AFM measurement are presented.

II. XRD analysis

The studied crystals have the preferred orientation associated with the (001) plane. On the obtained XRD spectra, in figure S1, the diffraction reflections from planes parallel to the surface, i.e. (002), (004), (006), and (0010), are shown. The angular position of these reflections depends on the sample composition, i.e., the content of Mo and W atoms, where pure $MoSe_2$ and WSe_2 samples determine the boundary values for every reflection. At the same time, the reflections obtained for $Mo_{1-x}W_xSe_2$ alloys are in the range determined by the mentioned boundary values. The width of this range is greater for the reflection from planes of a higher order, namely for (0010) one. To exclude the influence of the sample positioning related to the X-ray beam, all XRD spectra of $Mo_{1-x}W_xSe_2$ alloys were shifted to match the $MoSe_2$ (002) reflection position, i.e., to the 13.718 deg. This procedure will shift all reflections, but since these ones from planes of higher order are more widely spread, the influence of the composition of their position will be preserved. Finally, the composition of the samples was determined based on linear interpolation of the (0010) reflection between the position determined for the $MoSe_2$ and the WSe_2. This approach is justified because the distance between the $MoSe_2$ and WSe_2 reflections is less than 0.3 degrees, and hence we can assume that the sine function in the Bragg equation has a linear course over such a range.

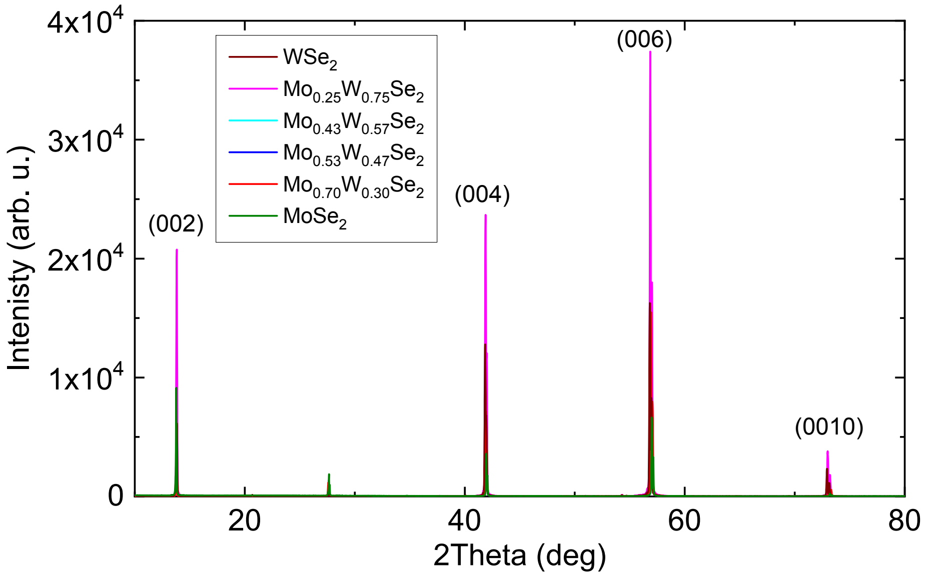

Figure S7. XRD spectra for studied samples – the diffraction reflections from planes (002), (004), (006), and (0010) are shown.

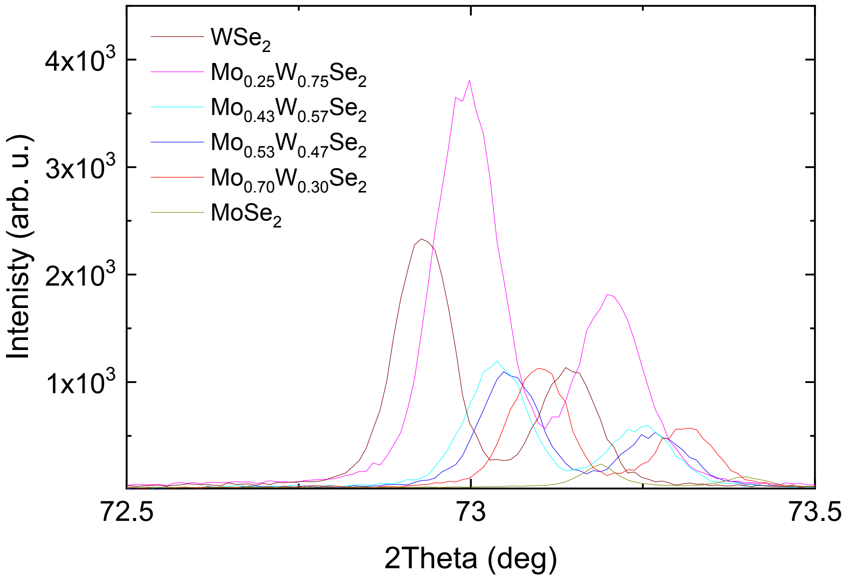

Figure S8. The detailed XRD spectra of (0010) plane reflections.

III. DFT calculations

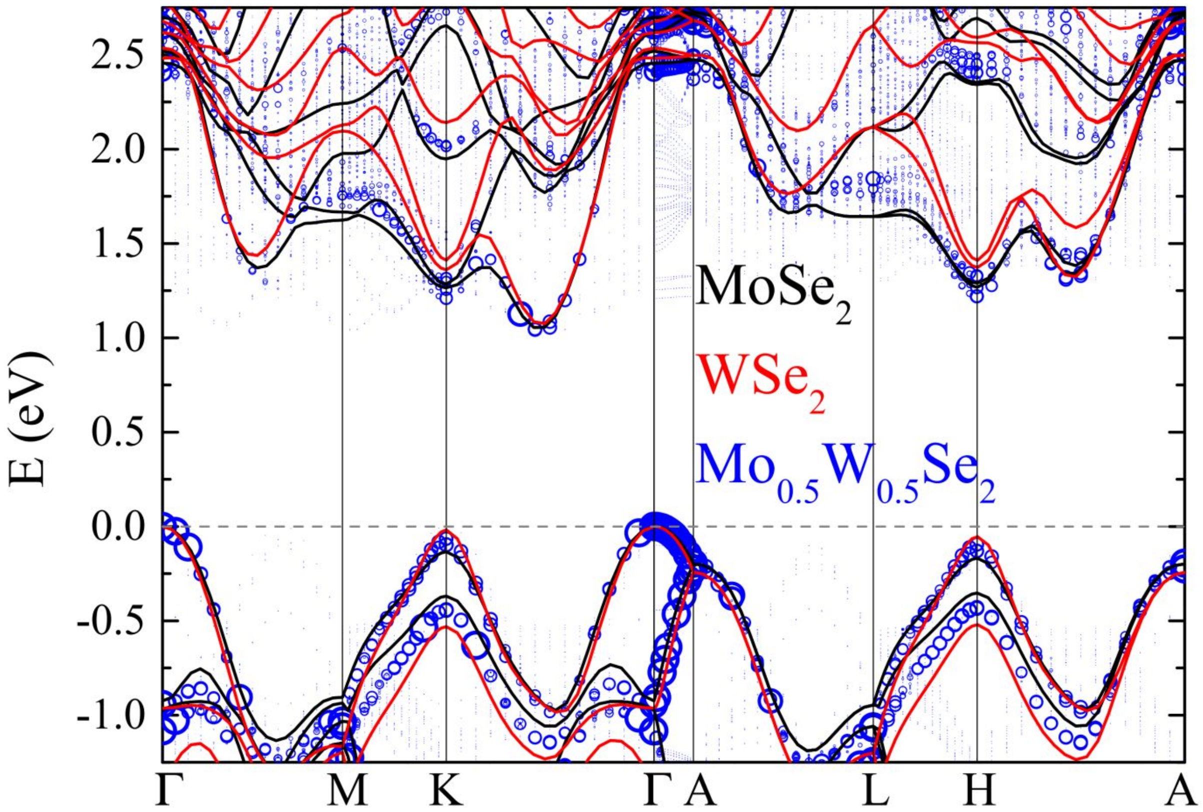

Figure S9. Band structures of $MoSe_2$ (black line), WSe_2 (red line) and $Mo_{0.5}W_{0.5}Se_2$ (blue circles). The size of the circle is proportional to the unfolding weight.
